# Reproducible Large-Scale Neuroimaging Studies with the OpenMOLE Workflow Management System

**DOI:** 10.3389/fninf.2017.00021

**Published:** 2017-03-22

**Authors:** Jonathan Passerat-Palmbach, Romain Reuillon, Mathieu Leclaire, Antonios Makropoulos, Emma C. Robinson, Sarah Parisot, Daniel Rueckert

**Affiliations:** ^1^BioMedIA Group, Department of Computing, Imperial College LondonLondon, UK; ^2^Institut des Systemes Complexes Paris Ile de FranceParis, France

**Keywords:** high performance computing, reproducibility, pipeline, large datasets, parameter exploration, neuroimaging, workflow systems

## Abstract

OpenMOLE is a scientific workflow engine with a strong emphasis on workload distribution. Workflows are designed using a high level Domain Specific Language (DSL) built on top of Scala. It exposes natural parallelism constructs to easily delegate the workload resulting from a workflow to a wide range of distributed computing environments. OpenMOLE hides the complexity of designing complex experiments thanks to its DSL. Users can embed their own applications and scale their pipelines from a small prototype running on their desktop computer to a large-scale study harnessing distributed computing infrastructures, simply by changing a single line in the pipeline definition. The construction of the pipeline itself is decoupled from the execution context. The high-level DSL abstracts the underlying execution environment, contrary to classic shell-script based pipelines. These two aspects allow pipelines to be shared and studies to be replicated across different computing environments. Workflows can be run as traditional batch pipelines or coupled with OpenMOLE's advanced exploration methods in order to study the behavior of an application, or perform automatic parameter tuning. In this work, we briefly present the strong assets of OpenMOLE and detail recent improvements targeting re-executability of workflows across various Linux platforms. We have tightly coupled OpenMOLE with CARE, a standalone containerization solution that allows re-executing on a Linux host any application that has been packaged on another Linux host previously. The solution is evaluated against a Python-based pipeline involving packages such as scikit-learn as well as binary dependencies. All were packaged and re-executed successfully on various HPC environments, with identical numerical results (here prediction scores) obtained on each environment. Our results show that the pair formed by OpenMOLE and CARE is a reliable solution to generate reproducible results and re-executable pipelines. A demonstration of the flexibility of our solution showcases three neuroimaging pipelines harnessing distributed computing environments as heterogeneous as local clusters or the European Grid Infrastructure (EGI).

## 1. Introduction

### 1.1. Problem

Larger sample sizes increase statistical power by reducing the variance of the sampling distribution. With large datasets like the Human Connectome Project^1^ (HCP) now freely available, one of the reasons why large studies are not more often conducted is the tremendous amount of computing power required. Distributed computing can offer this processing power but it can be hard to set up a distributed experiment for non-computer scientists.

Another important aspect to increase the quality and impact of scientific results is their capacity to be reproduced, especially by a different scientist. Researchers are more and more encouraged to share their experiments and the source code that led to the results they present. In order to be usable by other researchers, experiments have to be organized in a certain way.

Researchers are thus faced with two major problems in order to produce top quality studies: the necessity to provide a reproducible experimental protocol, and the technical challenge to upscale their implemented solutions to cope with large datasets. The whole solution must be made available in a relatively standard way so that other groups can pick up the experiment and re-run against their own set of resources and data.

What is the best way to describe experiments so that they can easily be reproduced by other researchers? Workflow, or pipelines, are a common way to model scientific problems involving different tools along multiple distinct stages. Although some initiatives try to unify workflow description (Amstutz et al., 2016), a majority of researchers still compose their pipelines using plain shell scripts. This approach makes it very hard to share the resulting pipelines, as shell scripts are strongly tied to their definition environment. Scripting languages are perfectly satisfying for workflow definition as long as they offer the readability and guided design that a high-level programming language does.

However, can we simply rely on a high-level scripting language to distribute the workload resulting from a pipeline? *Ad hoc* solutions to submit jobs to a local cluster are very efficient to quickly run an experiment. However, they cannot manage job resubmissions on unexpected failures, and are very unlikely to manage several computing environments. The resulting pipeline is once again not suitable to share with other researchers using another computing environment. A very good example in a widely distributed software package is FSL^2^ (FMRIB Software Library), which ships with pipelines that can only be delegated to a Sun Grid Engine (SGE) cluster.

Some applications might show more complicated than others to distribute in view of the complex set of dependencies they require for their execution. The DevOps community has tackled the problem of complex application deployments with an increasing use of software containers, the most famous solution being Docker. However, scientific computing environments are often designed as High Performance Computing (HPC) clusters, and cannot be customized for each user's needs. Cutting-edge containerization solution such as Docker are not available on these platforms, most of the time for security reasons as they require administrator privileges. While this is not a problem to empower the owner of a virtual machine with such privileges, HPC administrators are reluctant to grant such powers to researchers.

In order to build reproducible experiments at large scale, we thus need three elements:

- a simple access to large scale HPC/cloud environments
- a high-level formalism, such as workflows, to express the experiment in a portable way
- a standalone container platform that do not require administrator privileges at any point of its execution chain

In this paper, we introduce how the OpenMOLE (Reuillon et al., 2013) workflow management system can be paired up with the user-level archiver CARE (Janin et al., 2014) to address these problems in the context of large medical imaging studies.

### 1.2. Proposed solution

OpenMOLE is a generic workflow management solution not targeting a particular community. It allows users to embed their own application, rather than limiting them to a set of pre-packaged tools made available for a specific usage. Although this approach requires more involvement from the user's side, it also gives them more flexibility. Further down the line, a pipeline solution tailored for a specific field might not be suitable for multidisciplinary studies. In the specific case of neuroimaging projects, it is not rare to also collect genetics data in order to combine it with the information extracted from the images.

Reproducibility and sharing of OpenMOLE workflows start with its Domain Specific Language (DSL) that is used to describe the workflow steps and connections. The OpenMOLE DSL is an embedded DSL, written as a set of extensions to the Scala programming language. As a superset to Scala, it benefits from all the constructs available in this high-level programming language and harnesses Scala's strong type system to make workflow descriptions more meaningful and less error-prone. As a Scala application, OpenMOLE runs in the Java Virtual Machine (JVM) runtime. This makes it agnostic to its underlying Operating System (OS) and is another step toward sharing OpenMOLE workflows from one user to another, regardless of their work environment.

OpenMOLE is built with a strong focus toward the distribution of a pipeline workload to remote computing environments. Pipelines defined within the OpenMOLE framework are totally decoupled from the environments on which they are executed. This allows running the same pipeline on different environments without modifying the definition of the pipeline itself. On top of that, OpenMOLE was designed to enable a fine granularity of distribution. Individual tasks, or groups of tasks, can be deployed to different computing environments. This is particularly useful when a task of the pipeline requires specific devices such as GPUs to run, while the rest of the pipeline can be distributed to classic CPUs.

This work presents the integration of CARE archives as a new foundation to make tasks re-executable on the various computing environments supported by OpenMOLE. The CARE toolkit (Janin et al., 2014) provides a standalone containerization solution that does not need administrator privileges to re-execute on target hosts. While this perfectly fits our requirements for a solution in par with HPC environments' constraints, CARE cannot be used on its own to provide a standard format of exchange for scientific applications. It has not been built with this kind of applications in mind and focuses on providing low-level elements ensuring re-executability of a command line on any other Linux machine. However, its possibilities can be harnessed to form the base of a new OpenMOLE task re-executable on multiple environments.

Medical imaging pipelines are ideal candidates to evaluate our solution as they typically involve an heterogeneous software ecosystem. These software pieces usually come with a broad set of dependencies that are hard to track manually. They also manipulate large datasets that cannot be embedded in the software container and have to be transferred separately to the execution node running the current stage of the pipeline. The same remark applies to the pipeline's results as can be seen in Parisot et al. (2015) for instance.

### 1.3. Related work

#### 1.3.1. Generic workflow engines

Like OpenMOLE, other initiatives made the choice not to target a specific community. Kepler (Altintas et al., 2004) was one of the first general-purpose scientific workflow systems, recognizing the need for transparent and simplified access to high performance computing platforms more than a decade ago. Pegasus (Deelman et al., 2005) is a system that initially gained popularity for mapping complex workflows to resources resources in distributed environments without requiring input from the user.

PSOM (Pipeline System for Octave and Matlab) (Bellec et al., 2012) is a workflow system centered around Matlab/Octave. Although this is certainly a good asset for this userbase, it revolves around Matlab, a proprietary system. This hinders by definition sharing workflows to the wider community and reduces the reproducibility of experiments.

#### 1.3.2. Community-tailored workflow engines

On the other hand, some communities have seen the emergence of tailored workflow managers. For example, the bioinformatics community has developed Taverna (Oinn et al., 2004) and Galaxy (Goecks et al., 2010) for the needs of their community.

In the specific case of the neuroimaging field, two main solutions emerge: NiPype (Gorgolewski et al., 2011) and LONI (Rex et al., 2003). NiPype is organized around three layers. The most promising one is the top-level common interface that provides a Python abstraction of the main neuroimaging toolkits (FSL, SPM, …). It is extremely useful to compare equivalent methods across multiple packages. NiPype also offers pipelining possibilities and a basic workload delegation layer only targeting the cluster environments SGE and PBS. Workflows are delegated to these environments as a whole, without the possibility to exploit a finer grain parallelism among the different tasks.

The LONI Pipeline provides a graphical interface for choosing processing blocks from a predefined library to form the pipeline. It supports workload delegation to clusters preconfigured to understand the DRMAA API (Tröger et al., 2012).

However, the LONI Pipeline displays limitations at three levels. First, the format used to define new nodes is XML (eXtensible Markup Language), and assumes the packaged tools offer a well-formed command line and its input parameters. On this aspect, the Python interfaces forming NiPype's top layer is far superior to LONI pipeline's approach. Second, one might also regret the impossibility to script workflows, to the best of our knowledge.

The third and main drawback of the LONI pipeline is in our opinion its restrictive licensing, which prevents an external user to modify and redistribute the modifications easily. Previous works in the literature have shown the importance of developing and releasing scientific software under Free and Open Source licenses (Stodden, 2009; Peng, 2011). This is of tremendous importance to enable reproducibility and thorough peer-reviewing of scientific results.

Finally, we have recently noted another effort developed in Python: FastR^3^ (Achterberg et al., 2015). It is designed around a plugin system that enables connecting to different data sources or execution environments. At the moment, execution environments can only be addressed through the DRMA (Distributed Resource Management Application) API but more environments should be provided in the future.

#### 1.3.3. Level of support of HPC environments

Table 1 lists the support for various HPC environments in the workflow managers studied in this section. It also sums up the features and domains of application for each tool.

Table 1**Summary table of the features, HPC environments supported and domains of application of various workflow managers**.

**Table:** 

**Workflow engine**	**Local multi-processing**	**HPC support**	**Grid support**	**Cloud support**
Galaxy^4^	Yes	DRMAA clusters	No	No (manual cluster deployment)
Taverna^5^	Yes	No	No	No
FastR	Yes	DRMAA clusters	No	No
LONI^6^	No	DRMAA clusters	No	No (manual cluster deployment)
NiPype	Yes	PBS/Torque, SGE	No	No
Kepler^7^	Yes	PBS, Condor, LoadLeveler	Globus	No
Pegasus^8^	No (need local Condor)	Condor, PBS	No	No (manual cluster deployment)
PSOM	Yes	No	No	No
OpenMOLE	Yes	Condor, Slurm, PBS, SGE, OAR	*Ad hoc* grids, gLite/EMI, Dirac, EGI	EC2 (fully automated)^9^

**Table:** 

**Workflow engine**	**Scripting support**	**GUI**	**Generic/Community**	**License**
Galaxy	No	Yes	BioInformatics	AFL 3.0
Taverna	No	Yes	BioInformatics	Apache 2.0
FastR	Python	No	Neuroimaging	BSD
LONI	No	Yes	Neuroimaging	Proprietary (LONI)
NiPype	Python	No	Neuroimaging	BSD
Kepler	Partly with R	Yes	Generic	BSD
Pegasus	Python, Java, Perl	No	Generic	Apache 2.0
PSOM	Matlab	No	Generic	MIT
OpenMOLE	Domain Specific Language, Scala	Yes	Generic	AGPL 3

*Information was drawn from the web pages in footnote when present, or from the reference paper cited in the section otherwise*.

To the best of our knowledge, we are not aware of any workflow engine that targets as many environments as OpenMOLE, but more importantly that introduces an advanced service layer to distribute the workload. When it comes to very large scale infrastructures such as grids and clouds, sophisticated submission strategies taking into account the state of the resources as well as implementing a level of fault tolerance must be available. Most of the other workflow engines offer service delegation layers that simply send jobs to a local cluster. OpenMOLE implements expert submission strategies (job grouping, over submission, …), harnesses efficient middlewares such as Dirac, and automatically manages end-to-end data transfer even across heterogeneous computing environments.

Compared to other workflow processing engines, OpenMOLE promotes a zero-deployment approach by accessing the computing environments from bare metal, and copies on-the-fly any software component required for a successful remote execution. OpenMOLE also encourages the use of software components developed in heterogeneous programming languages and enables users to easily replace the elements involved in the workflow.

### 1.4. Main contributions

This paper puts the light on OpenMOLE's new features enabling large-scale pipelines to be reproducible while distributed to a large range of computing environments.

We first describe the three main elements from the OpenMOLE platform: (1) the DSL to design meaningful, reusable workflows, (2) the integration and simple access to a wide range of High Performance Computing (HPC) environments, and (3) the embedded parameter exploration methods (Section 2).

As evoked in the introduction, distributing an application can be troublesome. We list the potential issues encountered when distributing a typical medical imaging pipeline in Section 3. We then justify the solution chosen to enable re-executability and sharing of experiments in Section 3.2, and detail its implementation in OpenMOLE in Section 3.3.

This solution is evaluated with a workflow exploring the performance of different parameter initializations for decoding fMRI acquisitions from a canonical dataset (Haxby et al., 2001) (Section 4). The decoder is taken from the NiLearn tutorials (Abraham et al., 2014) and demonstrates how a workflow made of a complex combination of Python and native binary dependencies can be successfully reproduced on different computing platforms without any prior knowledge regarding the state of their software stack. This study demonstrates the potential of this work to process a well-known dataset for which the performance and validity of the pipeline can be evaluated.

As a case-study, we finally detail three neuroimaging pipelines managed by OpenMOLE and the different benefits brought by the platform and its software ecosystem (Section 5).

## 2. What is OpenMOLE?

Scientific experiments are characterized by their ability to be reproduced. This implies capturing all the processing stages leading to the result. Many execution platforms introduce the notion of workflow to do so (Barker and Van Hemert, 2008; Mikut et al., 2013). Likewise, OpenMOLE manipulates workflows and distributes their execution across various computing environments.

A workflow is a set of tasks connected through transitions. From a high level point of view, tasks comprise inputs, outputs and optional default values. Tasks describe what OpenMOLE should execute and delegate to remote environments. They embed the actual applications to study. Depending on the kind of program (binary executable, Java…) to embed in OpenMOLE, the user chooses the corresponding task. Tasks execution depends on inputs variables, which are provided by the dataflow. Each task produces outputs returned to the dataflow and transmitted to the input of consecutive tasks. OpenMOLE exposes entry points to inject data in the dataflow (*sources*) and extract useful results at the end of the experiment (*hooks*).

**Figure 1:**
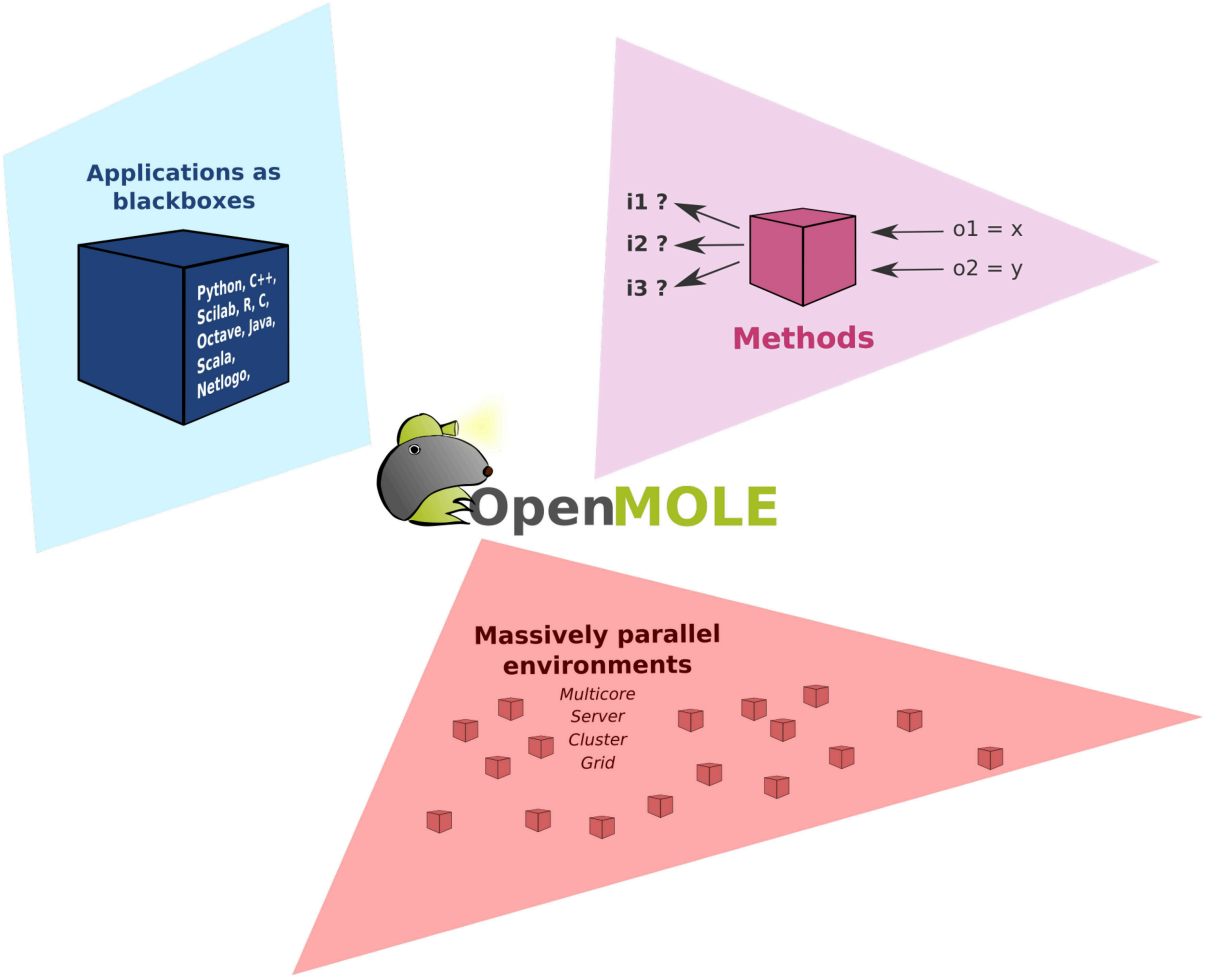
**Organization of OpenMOLE around three axes: the ***Applications***, the exploration ***Methods*** and the support of ***Massively parallel environments*****.

As shown in Figure 1, OpenMOLE revolves around three main elements: the *Applications*, the exploration *Methods* and the support of *Massively parallel environments*. These three components are put together in a common DSL to describe the workflows.

We will give a quick overview of these different components in the subsections. For more details regarding the core implementation and features of OpenMOLE, interested readers can refer to Reuillon et al. (2010, 2013, 2015a) and the OpenMOLE website (Reuillon et al., 2015b).

### 2.1. A DSL to describe workflows

According to Barker and Van Hemert (2008), workflow platforms should not introduce new languages but rely on established ones. OpenMOLE's DSL is based on the high level Scala programming language (Odersky et al., 2004).

OpenMOLE's DSL introduces new operators in the Scala programming language to manage the construction and execution of the workflow. The advantage of this approach lies in the fact that workflows can exist even outside the OpenMOLE environment. As a high-level language, the DSL can be assimilated to an algorithm described in pseudo-code, easily understandable by most scientists. Moreover, it denotes all the types and data used within the workflow, as well as their origin. This reinforces the capacity to reproduce workflow execution both within the OpenMOLE platform or using another tool.

The philosophy of OpenMOLE is *test small* (on a local computer) and *scale for free* (on remote distributed computing environments). The DSL supports all the Scala constructs and provides additional operators and classes especially designed to compose workflows. OpenMOLE workflows expose implicit parallel aspects of the workload that can be delegated to distributed computing environments in a transparent manner.

### 2.2. Distributed computing environments

OpenMOLE helps delegate the workload to a wide range of HPC environments including remote servers (through SSH), clusters (supporting the job schedulers PBS, SGE, Slurm, OAR, and Condor), computing grids running the gLite/EMI middleware (through the WMS, CREAM and DIRAC entry points) and Amazon Elastic Compute Cloud (EC2). Support to these environments is implemented in GridScale^10^, a Free and Open Source Scala library.

Building on top of GridScale's as a service layer, OpenMOLE's simple workflow description is quite convenient to determine the computing environment best suited for a workflow. Switching from one environment to another is achieved by modifying a single line in the script. The granularity of the implementation allows each task of the workflow to be assigned to a different execution environment. This feature proves very useful when considering the limited availability of a particular resource (shared cluster) or its suitability to process a particular problem (necessity to be processed on a GPU or another type of hardware accelerator).

The final workflow description can thus connect tasks using different software components but also running on heterogeneous execution environments thanks to GridScale's large support of HPC platforms.

The execution platform of OpenMOLE has proved to be robust enough to manage no less than half a billion instances (Schmitt et al., 2015) of a task delegated to the European Grid Infrastructure (EGI).

### 2.3. Exploration methods

OpenMOLE has been designed with distributed parameter space exploration as a core use case (Reuillon et al., 2013). First its DSL comprehends a high level representation of design of experiments^11^, which is concise and expressive. For instance expressing the exploration a full-factorial combination on a discrete parameter *i*, a continuous one *x*, a set of files *f* in a directory and replicate the experiment 10 times with randomly generated seeds *s* is expressed as shown in Listing 1:

**Listing 1:**
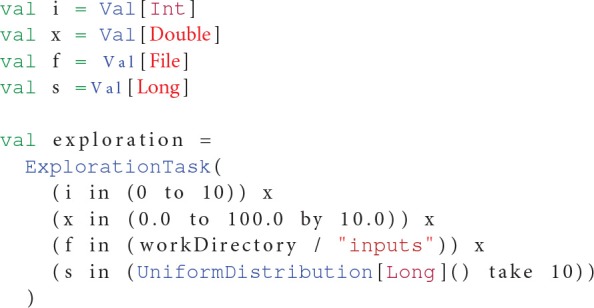
**Sampling example in OpenMOLE**.

OpenMOLE also proposes advanced design of experiments with better coverage properties such as the low discrepancy Sobol sequence^12^ and the Latin Hypercube Sampling (LHS)^13^. These sampling methods have been widely uses for model exploration and are also adapted to evaluate other classes of parametric algorithms.

In addition to these classical a priori sampling methods, OpenMOLE generic formalism is a prolific playground to develop innovative exploration methods based on iterative refinement of the sampling. In these methods the results (*outputs*) of the explored program are taken into account in order to generate additional samples at interesting locations in the parameter space. These exploration methods are aimed to better comprehend the behavior of an application, or to finely tune parameters.

Several state-of-the art iterative methods have been developed, evaluated and made available through OpenMOLE (multi-objective calibration (Schmitt et al., 2015), calibration profile (Reuillon et al., 2015c), Pattern Space Exploration Chérel et al., 2015; Cottineau et al., 2015a) and more are being developed such as the model family method (Cottineau et al., 2015b). Implementations of Evolutionary Algorithms (EA) techniques taken from the literature such as (Deb et al., 2002) are also available.

Integrating these methods into OpenMOLE makes them available to a wide range of use cases (modeling, algorithm benchmarking, parameter tuning and testing applications…). The methods pair up perfectly with OpenMOLE as they are inherently parallel algorithms that can be distributed. The exploration methods elements of OpenMOLE thus benefit from the wide range of distributed computing environments available in the platform.

## 3. The challenges of distributing applications

### 3.1. Problems and classical solutions

Let us consider all the dependencies introduced by software bundles explicitly used by the developer. They can take various forms depending on the underlying technology. Compiled binary applications will rely on shared libraries, while interpreted languages such as Python will call other scripts stored in packages.

These software dependencies become a problem when distributing an application. It is very unlikely that a large number of remote hosts are deployed in the same configuration as a researcher's desktop computer. Actually, the larger the pool of distributed machines, the more heterogeneous they are likely to be.

If a dependency is missing at runtime, the remote execution will simply fail on the remote hosts where the requested dependencies are not installed. An application can also be prevented from running properly due to incompatibilities between versions of the deployed dependencies. This case can lead to silent errors, where a software dependency would be present in a different configuration and would generate different results for the studied application.

Silent errors break Provenance, a major concern of the scientific community (Miles et al., 2007; MacKenzie-Graham et al., 2008). Provenance criteria are satisfied when an application is documented thoroughly enough to be reproducible. This can only happen in distributed computing environments if the software dependencies are clearly described and available.

Some programming environments provide a solution to these problems. Compiled languages such as C and C++ offer to build a static binary, which packages all the software dependencies. Some applications can be very difficult to compile statically. A typical case is an application using a closed source library, for which only a shared library is available.

Another approach is to rely on an archiving format specific to a programming language. The most evident example falling into this category are Java Archives (JAR) that embed all the Java libraries an application will need.

A new trend coming from recent advances in the software engineering community is embodied by Docker. Docker has become popular along with other DevOps techniques to improve efficiency of software engineers. It enables shipping an application within a so-called container that will include the application and its required set of dependencies. Containers can be transferred just like an archive and re-executed on another Docker engine. Docker containers run in a sandboxed virtual environment but they are not to be confound with virtual machines. They are more lightweight as they don't embed a full operating system stack. The use of Docker for reproducible research has been tackled in Boettiger (2014) and Chamberlain et al. (2014).

The main drawback of Docker is that it implies deploying a Docker engine on the target host. Having a Docker engine running on every target host is an unlikely hypothesis in heterogeneous distributed environments such as computing grids. It is also impossible to deploy a Docker engine on the fly as its execution requires administrator privileges. Such privileges are not granted to end-users on HPC infrastructures at the heart of most scientific computing experiments. This is only the case in a fully-controlled environment, most of the time a cloud-based deployment where the user controls his own virtual machines.

The last option is to rely on a third-party application to generate re-executable applications. The strategy consists in collecting all the dependencies during a first execution in order to store them in an archive. This newly generated bundle is then shipped to remote hosts instead of the original application. This is the approach championed by tools like CDE (Guo, 2012), ReproZip (Chirigati et al., 2013), or CARE (Janin et al., 2014).

Considering all these aspects, the OpenMOLE platform has for long chosen to couple with tools providing standalone packages. While CDE was the initial choice, recent requirements in the OpenMOLE user community have led the development team to switch to the more flexible CARE. The next section will detail why OpenMOLE relies on CARE to package applications.

### 3.2. Why should i CARE?

The first step toward spreading the workload across heterogeneous computing elements is to make the studied application executable on the largest number of environments. We have seen previously that this could be difficult with the entanglement of complex software environments available nowadays. For instance, a Python script will run only in a particular version of the interpreter and may also make use of binary dependencies. The best solution to make sure the execution will run as seamlessly on a remote host as it does on the desktop machine of the scientist is to track all the dependencies of the application and ship them with it on the execution site.

OpenMOLE used to provide this feature through a third-party tool called CDE (Code, Data, and Environment packaging) (Guo, 2012). CDE creates archives containing all the items required by an application to run on any recent Linux platform. To do so, it tracks all the files that interact with the application and creates the base archive. At the time of writing, CDE appears not to be maintained anymore, the last significant contribution to the main source tree dating back from 2012^14^.

The only constraint regarding CDE is to create the archive on a platform running a Linux kernel from the same generation as those of the targeted computing elements. As a rule of thumb, a good way to ensure that the deployment will be successful is to create the CDE package from a system running Linux 2.6.32. Many HPC environments run this version, as it is the default kernel used by science-oriented Linux distribution, such as Scientific Linux and CentOS.

CARE on the other hand presents more advanced features than CDE. CDE actually displays the same limit than a traditional binary run on a remote host: i.e., the archive has to be generated on a platform running an old enough Linux kernel, to have a maximum compatibility with remote hosts. CARE lifts this constraint by emulating missing system calls on the remote environment. Thus, an application packaged on a recent release of the Linux kernel will successfully re-execute on an older kernel thanks to this emulation feature. CARE is, to the best of our knowledge, the only standalone solution ensuring re-execution on any Linux host, regardless of the original packaging host and without requiring administrator privileges.

We have also noted ReproZip (Chirigati et al., 2013) as a promising packaging solution. ReproZip's most interesting feature is to produce a package that can be re-run against different backends. Standalone archives can be extracted as plain folders, and then re-executed in a chrooted environment using the target host's environment and installed packages. Another option is to install them in the host system as a package in the case of a Debian-based Operating System. Although they don't require any pre-installed software, these solutions cannot ensure a successful re-execution due to low-level incompatibilities between the packaging and extraction environments. Other extraction solutions for ReproZip offer to run in a Vagrant virtual machine or a Docker container. However, none of these solution fit our design assumptions to exploit arbitrary environments without having to deploy anything beforehand.

The next section will describe how OpenMOLE integrates CARE seamlessly, as a first-class citizen in the DSL.

### 3.3. Combining OpenMOLE with CARE

Different types of tasks co-exist in OpenMOLE workflows, each embedding a different kind of application. Portable applications packaged with CARE are handled by the CARETask. Packaging an application is done once and for all by running the original application against CARE. CARE's re-execution mechanisms allow changing the original command line when re-running an application. This way we can update the parameters passed on the command line and the re-execution will be impacted accordingly. As long as all the configuration files, libraries, and other potential dependencies were used during the original execution, there is no need to package the application multiple times with different input parameters. To ensure all the initial execution conditions are captured, the environment variables defined in the session are also stored in the archive and populated on re-execution.

The newly packaged archive is the first argument expected by the CARETask. The second argument corresponds to a modified command line, updating the original call to specify a different parameter combination for each instance. The CARETask performs two actions: it first extracts the CARE archives by executing *archive.tgz.bin* (the archive is a self-extracting executable). The actual re-execution can then take place in the freshly unarchived work directory. Note that for each execution of the CARETask, any command starting with/is relative to the root of the CARE archive's filesystem, and any other command is executed in the current directory. The current work directory defaults to the original packaging directory.

**Figure 2:**
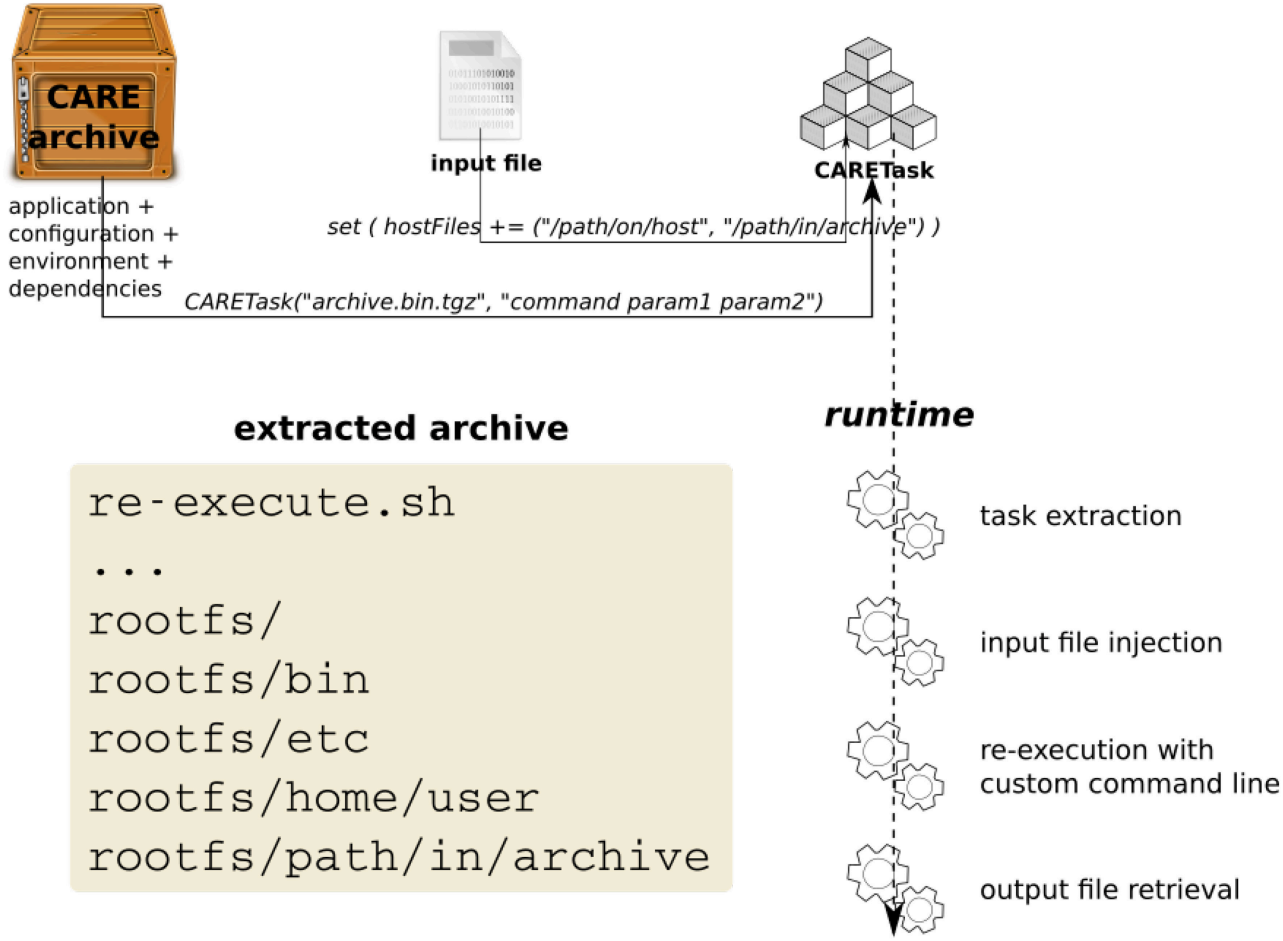
**Embedding a CARE archive in OpenMOLE with the CARETask**.

Figure 2 represents the interactions between the CARE archive and the CARETask in OpenMOLE.

The CARETask can be customized to fit the needs of a specific application. For instance, some applications disregarding standards might not return the expected 0 value upon successful completion. The return value of the application is used by OpenMOLE to determine whether the task has been successfully executed, or needs to be re-executed. Setting the boolean flag errorOnReturnValue to false will prevent OpenMOLE from re-scheduling a CARETask that has reported a return code different from 0. The return code can be saved in a variable using the returnValue setting.

Another default behavior is to print the standard and error outputs of each task in the OpenMOLE console. Such raw prints might not be suitable when a very large number of tasks is involved or that further processing are to be performed on the outputs. A CARETask's standard and error outputs can be assigned to OpenMOLE variables and thus injected in the dataflow by summoning respectively the stdOut and stdErr actions on the task.

When packaging an application with CARE, we make sure of excluding any input data from the archived files. CARE allows this with the option -p. Data can later be reinjected in the archive from OpenMOLE using the inputFiles directive. This directive accepts OpenMOLE variables that describe a set of files to be used as parameters. This means that each instance of a CARETask will see a different input data in its archive's filesystem. The task instance's work directory will thus contain the extracted application supplemented by the specific input data files that were previously discarded from the packaging stage. In this configuration, input data are perfectly decoupled from the application and can be manipulated using OpenMOLE's advanced parameter exploration methods, before being injected to the appropriate task.

Files that are not part of the exploration can also be made available within the CARETask's filesystem using either the hostFiles or resources directives.

Listing 2 demonstrates the elements of the CARETask described in this section.

**Listing 2:**
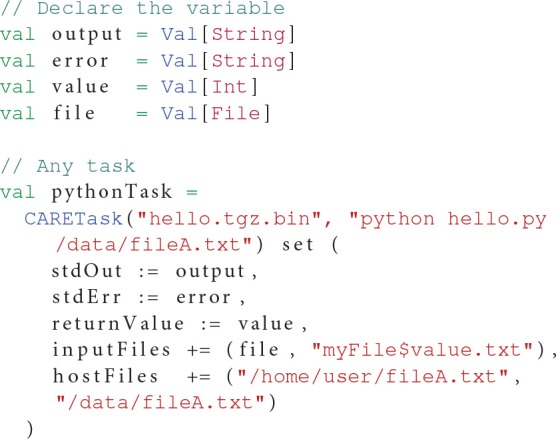
**Example of a CARETask using a file from the host injected in the archive**.

The support of CARE as a first-class citizen in the platform added to existing OpenMOLE features enforces provenance in workflows at two levels. Not only the workflows are defined using a platform agnostic language, but we can now ship standalone archives containing re-executable applications for each stage of the pipeline.

Integrating CARE in OpenMOLE has enhanced the scope of potential applications for CARE, which was initially designed as a tool to create comprehensive bug reports. The development efforts made in OpenMOLE over the past few months have propelled CARE in the range of potential solutions to enable reproducibility in scientific experiments. This integration layer was necessary to bridge the gap between CARE and the scientific community, in order to provide a simple interaction with the end-user.

The next section will show how the CARETask can help explore a canonical dataset on a heterogeneous set of computing infrastructures, and create a reproducible workflow describing the experiment.

## 4. Evaluation of the reproducibility of a neuroimaging workflow

We will evaluate the reproducibility enabled by the CARETask using an fMRI decoder on the Haxby dataset (Haxby et al., 2001). The goal of this experiment is to show that a pipeline intended to run on a local machine and requiring a set of preinstalled dependencies can be re-executed on various distributed computing environments using the CARETask. It validates the choice of the CARE technology to package applications and demonstrates the OpenMOLE integration that enables CARE to be used to reproduce scientific experiments.

### 4.1. Parameter space exploration of a classifier

This experiment is based on a tutorial ^15^ for the NiLearn package (Abraham et al., 2014). The example compares different classifiers on a visual object recognition decoding task using the Haxby dataset (Haxby et al., 2001).

The Haxby dataset consists in the fMRI activity recorded for 6 subjects exposed to various stimuli from different categories. The example evaluates the performance of different parameter initialization of a logistic regression classifier to predict the category the subject is seeing from the fMRI activity. Significant prediction shows that the signal in the region contains information about the corresponding category.

We have slightly modified the online example to focus on well-known classifier: the logistic regression. In the NiLearn tutorial, two input parameters vary for this algorithm. The same parameter ranges are tested for this classifier as detailed in Table 2. In order to obtain comparable results, we have set the seed of the pseudorandom number generator used in the logistic regression to 0.

**Table 2:** **Parameters and their values for the Logistic Regression classifier**.

**Parameter**	**Range**	**Description**
C	{0.1; 0.5; 1; 5; 10; 50; 100}	Inverse of regularization strength
Penalty	{11; 12}	Norm used in the penalization
Seed	0	Seed initializing the Pseudorandom number generator

**Figure 3:**
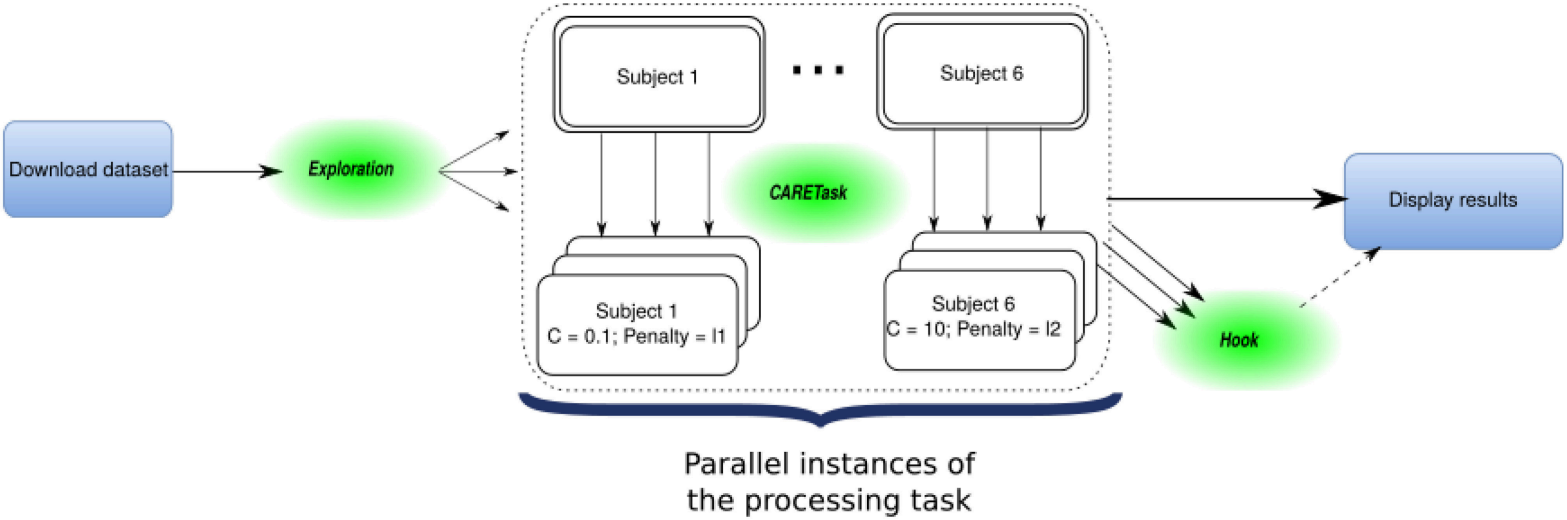
**Representation of the Haxby decoder workflow: OpenMOLE elements (***Exploration***, ***Hook***) are intertwined with the native pipeline's steps (***download***, ***processing***, ***display results***) to form the whole parallel workflow**.

The OpenMOLE workflow for this experiment is made of multiple tasks running both locally and on remote execution nodes as depicted in Figure 3. The initial task asks NiLearn to download the whole dataset from an online repository. An ExplorationTask then determines the parameter space that will be explored in parallel by OpenMOLE. The processing task takes a specific tuple of initialization parameters for the logistic regression from the exploration, along with a single subject as in the original example. Each instance of the processing task computes a leave-one-out cross-validated score for the logistic regression classifier initialized with the given input parameters. Result files are retrieved using the OpenMOLE hook mechanism from the remote execution node. They contain a serialized data structure with the results of the processing task stored in Python's pickle format. The collected results are aggregated on the host machine and plotted locally in a separate PNG file per subject. Input and result files are automatically transferred and passed to the next task, regardless of their format by OpenMOLE's internal mechanisms.

### 4.2. Testing the reproducibility

The experiment aims at testing the reproducibility of the whole workflow on each of the platforms described in Table 3. The workflow is considered successfully reproduced when generating the exact same result from one machine to another. This for two reasons:

- The seed of the PseudoRandom Number Generator (PRNG) was set to the same value (0) for each instance of the parameter exploration and across the execution environments. This disables any stochastic variability in the results;
- The floating precision reported in the original version of the tutorial is low enough (two digits) so that the underlying hardware does not impact the final results.

Table 3**Different configurations employed in the reproducibility experiment**.

**Table:** 

**Denomination**	**Resource manager/Scheduler**	**CPUs**	**Execution time**	**Operating system**	**Linux kernel**
*Personal machine*	None	4 cores	20′36″	Debian 8	4.6.0-1-amd64
*Desktop machine*	SSH	8 cores	28′14″	Ubuntu 14.04	3.13.0-91-generic
*Lab′s private cluster*	Slurm	312 cores	14′50″	Ubuntu 14.04	3.13.0-63-generic
*College wide cluster*	PBS	13,558 cores	48′25″	Red Hat Enterprise Linux Server release 6.7	2.6.32-573.12.1.el6.x86_64
*European Grid Infrastructure (EGI)*	EMI/gLite	650,000 cores	27′15″	CentOS 6/Scientific Linux	2.6.32-642.6.2.el6.x86_64

**Table:** 

**Denomination**	**File system**	**Python version**	**Java runtime environment**	**Administrator privileges**
*Personal machine*	Permanent	2.7.12	OpenJDK 1.8.0_91	Yes
*Desktop machine*	Shared, permanent	2.7.6	OpenJDK 1.7.0_101	Yes
*Lab's private cluster*	Shared, permanent	2.7.6	OpenJDK 1.7.0_101	No
*College wide cluster*	Temporary	2.6.6	OpenJDK 1.7.0_101	No
*European Grid Infrastructure (EGI)*	Shared, temporary	2.7.8	OpenJDK 1.6.0_40	No

The ensemble of Python scripts taken from the NiLearn tutorial to form the workflow steps were packaged as a single CARE archive on the host labeled *Personal machine* in Table 3. There is no need to know about the packaged tool in details, or to manually track its software dependencies. Only the input and output data (results) locations must be known so that they can be excluded from the archive. Input data and results are dynamically injected and extracted at runtime from and to the OpenMOLE dataflow. This perfectly fits OpenMOLE's definition of a workflow as a set of connected black boxes only communicating with the external world through their inputs and outputs.

The archive embeds the following Python packages installed in a virtual environment along with their own binary dependencies:

- matplotlib (1.5.1)
- nibabel (2.0.2)
- nilearn (0.2.5)
- numpy (1.11.1)
- pip (8.1.2)
- scikit-learn (0.17.1)
- scipy (0.17.1)
- virtualenv (15.0.2)

The only common aspect between the platforms in Table 3 is that their Operating System (OS) runs Linux as a kernel.

The heterogeneity in Java Runtime Environment (JRE) versions is solved by OpenMOLE shipping with its own JRE (OpenJDK 1.8.0) to execute on remote machines. It has been built against a 2.6.32 Linux kernel in order to ensure it re-executes successfully on the largest possible range of Linux platforms.

The execution time is only reported here as a marker of successful re-execution on the given platform. Multiple parameters can explain the variability from one environment to another, the most obvious being the different availability of the required resources.

Table 4 reports the prediction scores resulting from running the pipeline on the first subject of the dataset. The prediction scores obtained are similar to those obtained in the tutorial for equivalent parameters (ex: *C* = l1, *p* = 50), down to the second decimal.

**Table 4:** **Average prediction scores out of 12 leave-one-out cross validations (± standard deviation) for subject 1 of the Haxby dataset**.

**Penalty**	**C**	**Bottle**	**Cat**	**Chair**	**Face**	**House**	**Scissors**	**Scrambledpix**	**Shoe**
	*000.10*	0.175096102728	0.379731749159	0.158451539359	0.604156173217	0.848084821382	0.233506609807	0.666680287676	0.375028443778
		(±0.231049939141)	(±0.276586523275)	(±0.164116793689)	(±0.230013422495)	(±0.193749012866)	(±0.244159557883)	(±0.147995188894)	(±0.269241140013)
	*000.50*	0.451689065765	0.62791249587	0.406748456287	0.732189944558	0.87213622291	0.44593597263	0.716080161668	0.493902257873
*l1*		(±0.150073309605)	(±0.244023131922)	(±0.13915694051)	(±0.16331860702)	(±0.173805716785)	(±0.257571429727)	(±0.174958432456)	(±0.193535971087)
	*001.00*	0.47399243887	0.632733430141	0.440552878726	0.734600107495	0.891882988013	0.429409863592	0.703740403044	0.487136011563
		(±0.148313849961)	(±0.22733488965)	(±0.137281494554)	(±0.133217998162)	(±0.133412666949)	(±0.28355944954)	(±0.175397314428)	(±0.18609138877)
	*005.00*	0.471410676788	0.619848767217	0.445594322982	0.73255493045	0.888374216083	0.432704249094	0.69471103372	0.482796210813
		(±0.164574051317)	(±0.219134957488)	(±0.0886794828788)	(±0.118718190623)	(±0.134171749036)	(±0.268164483186)	(±0.203315689402)	(±0.189916530226)
	*010.00*	0.466838744958	0.638928250112	0.444088450235	0.732606643443	0.892668605904	0.405431521822	0.71285195265	0.497537256804
		(±0.166579054815)	(±0.219737279322)	(±0.0842821140037)	(±0.122777408669)	(±0.118406687757)	(±0.281777460975)	(±0.210434007693)	(±0.192609613885)
	*050.00*	0.489227398669	0.63862354636	0.455303030303	0.688123601676	0.857546710256	0.416753246753	0.764532755937	0.486474730818
		(±0.173950833724)	(±0.182743896393)	(±0.120853053014)	(±0.109180148231)	(±0.117807127625)	(±0.263269896373)	(±0.201940516096)	(±0.18853099241)
	*100.00*	0.478975007701	0.673136147956	0.478630692661	0.648015275109	0.830941774901	0.437724466891	0.755797787415	0.495224735512
		(±0.188926437971)	(±0.164701994218)	(±0.156000687925)	(±0.157277843991)	(±0.166878589573)	(±0.231837298803)	(±0.208210996079)	(±0.181335061366)
	*000.10*	0.419064747547	0.529790472655	0.540197885259	0.524839160021	0.607328524302	0.503213203538	0.775192036147	0.511069835451
		(±0.196075499695)	(±0.214583690108)	(±0.177491061481)	(±0.174635484257)	(±0.219564887192)	(±0.157493925525)	(±0.182461202755)	(±0.190309164145)
	*000.50*	0.442440703126	0.541560090043	0.545476902154	0.540376138138	0.633534946986	0.514000952751	0.790346387359	0.492847276932
*l2*		(±0.20440149609)	(±0.206602126667)	(±0.17485799816)	(±0.184370175442)	(±0.230112025184)	(±0.151190492209)	(±0.185784023473)	(±0.165133338032)
	*001.00*	0.43321401391	0.5356102353	0.539036006956	0.549934036724	0.63394509057	0.511795894766	0.779956776969	0.506150386029
		(±0.196947156928)	(±0.203785520359)	(±0.168881360193)	(±0.188614078434)	(±0.223754917472)	(±0.147923692585)	(±0.181218528886)	(±0.168495305593)
	*005.00*	0.437276734917	0.539067074353	0.536326639695	0.561171120546	0.639533423003	0.51426105273	0.772198269399	0.505117174666
		(±0.19981805444)	(±0.204503694746)	(±0.178953853726)	(±0.199103541317)	(±0.224359546867)	(±0.152538374463)	(±0.182761066327)	(±0.166622660548)
	*010.00*	0.436835824451	0.53423394452	0.535061891372	0.563763615878	0.639533423003	0.514457769593	0.769480878095	0.507298139645
		(±0.200058165728)	(±0.204880105531)	(±0.177213495836)	(±0.199772413453)	(±0.224359546867)	(±0.154085991243)	(±0.182828486106)	(±0.166133356219)
	*050.00*	0.438753630834	0.542474425035	0.531695098097	0.561135198439	0.644376756606	0.495566262135	0.769480878095	0.504692100888
		(±0.206114028623)	(±0.21163564676)	(±0.174964145627)	(±0.200185974598)	(±0.226195394616)	(±0.144364998604)	(±0.182828486106)	(±0.167107109882)
	*100.00*	0.438753630834	0.546178279103	0.530134975891	0.561135198439	0.643647391194	0.495953500594	0.764509986917	0.503501624698
		(±0.206114028623)	(±0.206459632778)	(±0.173661532191)	(±0.200185974598)	(±0.226021956671)	(±0.144993468122)	(±0.183050686489)	(±0.167085408715)

An even more interesting aspect of this technique is that we obtained identical results from one environment to another, across all the platforms described in Table 3. In order to switch the execution of the processing task from one environment to another, only one line was impacted in the workflow. File transfers are managed by OpenMOLE as well as data injection at the right location of the CARE pseudo file system. This is shown in Listing 3 and is further detailed in specific case studies in Sections 5.1 and 5.2.

**Listing 3:**
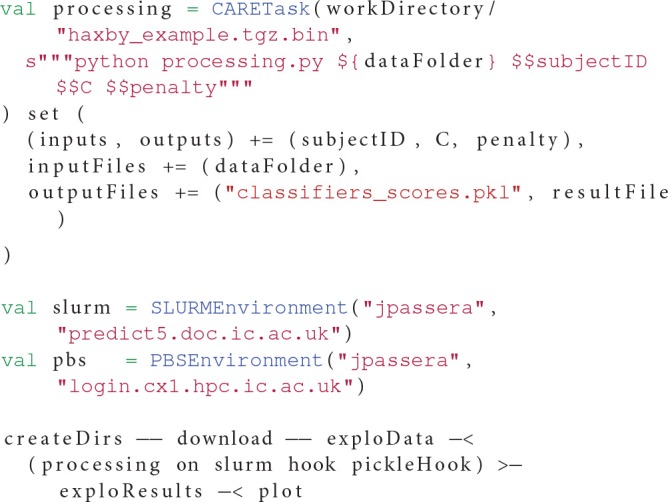
**Data injection and environment switching in the Haxby workflow**.

This experiment demonstrated OpenMOLE's ability to efficiently delegate the workload of a real-world pipeline to an heterogeneous set of computing environments. Coupling CARE and OpenMOLE in the CARETask enables experiments to be designed on a personal machine using any toolkit or programming language. Experiments can then be distributed to remote environments regardless of the availability of the tools they depend on, or the ability of the user to install new software components on the target environment (as illustrated by the *Administrator Privileges* column in Table 3).

On a side note, this experiment has shown that the genericity of the OpenMOLE platform was not a barrier to exploit field-specific tools in a workflow, NiLearn in this case. By focusing on providing a high-level workflow formalism and simplifying the access to HPC environments, this experiment has shown OpenMOLE was flexible enough to address the needs of the neuroimaging community while respecting their popular software ecosystem.

Finally, this experiment has highlighted the role the CARETask could play in producing reproducible results and re-executable pipelines. Section 5 will now feature the CARETask in combination with the DSL and various computing environments throughout three real-world examples of neuroimaging pipelines.

## 5. Case studies

The source code and required material for the three case studies is not part of the OpenMOLE market place^16^ due to license restrictions induced by some of the binary dependencies. It is however available in its own repository^17^ and contains entries presented as they would be on the original market place. For the sake of clarity, this section will only highlight the parts relevant with the use case.

### 5.1. Multiple environments in the same workflow

The first workflow preprocesses the input data as necessary for a brain parcellation algorithm. Brain parcellation is an essential task for the construction of brain connectivity networks, which has the potential to provide new insights into the brain's organization. Brain parcellation aims at regrouping brain regions that have similar connectivity profiles to the rest of the brain, so as to construct connectivity networks of tractable dimension for subsequent analysis.

The method proposed in Parisot et al. (2015) uses diffusion Magnetic Resonance Imaging (dMRI) data and structural connectivity to drive the parcellation task. dMRI provides an indirect measurement of the brain's structural connectivity (white matter fiber tracts), by measuring the anisotropy of water molecules in the brain. Several processing steps are required in order to recover the white matter tracts and consequently parcellate the brain from dMRI volumes. In Parisot et al. (2015), the data is processed using FSL's bedpostX and probtrackX (Behrens et al., 2007; Jbabdi et al., 2012), which estimate the fibres orientations at each voxel and perform probabilistic tractography respectively. Both methods are very time consuming. On high quality data such as the HCP database ^18^, BedpostX takes approximately a week on CPU and 3 h on GPU, while ProbtrackX runs for approximately 30 h. In order to process a large group of subjects for group-wise analysis in a reasonable amount of time, it is necessary to use BedpostX's GPU-enabled version (Hernández et al., 2013) and process the subjects in parallel.

This workflow benefits from OpenMOLE's capacity of delegating different tasks of the pipeline to different computing environments. In this workflow, the first tasks runs a GPU-enabled version of the FSL bedpostX tool (Hernández et al., 2013) while the rest of the workflow is executed on CPU. We thus leverage two distinct computing environments to delegate the workload of this workflow. Listing 4 highlights the section of the workflow description declaring two environments and connecting them with the corresponding tasks.

**Listing 4:**
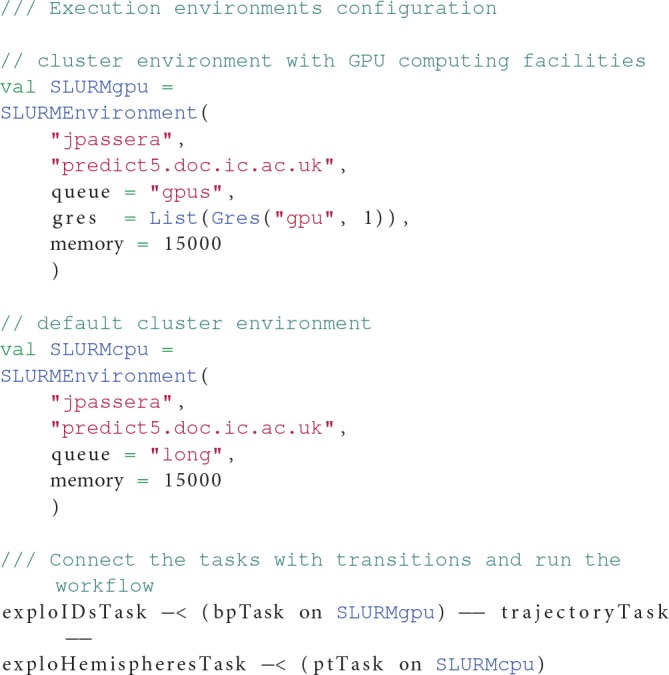
**Multiple environments used by the parcellation preprocessing workflow. The bpTask task requires a GPU to run so it is assigned to the *SLURMgpu* environment, whereas pbTask can run on traditional CPUs. Both *SLURMxxx* environments are ubiquitous declinations of the same Slurm cluster, with different requirements**.

It is worth noting that the required authentications to connect to the environment do not have to appear in the workflow description, but are specified once and for all to the platform. Authentications are from then on encrypted and stored in the user's preferences folder.

It is valid in the OpenMOLE syntax for the same remote host to appear in different environment blocks. This ubiquity in environments enables specifying different settings for the same computing host, for example different memory requirements, or devices in the present case. This feature goes along with the ability of each task to run on a separate environment to increase the finer parallelism granularity in the workflow.

Environments are only associated with the tasks at the final stage of the workflow description when tasks are also interconnected. The workflow could be shared without the environments and remain syntactically correct. Users familiar with other computing environments can simply replace the environment declaration by the one of their choice, all in a single location.

### 5.2. Sharing a pipeline with the community

The second workflow in this study segments a collection of developing brain images using the Draw-EM software. Draw-EM^19^ (Developing brain Region Annotation With Expectation-Maximization) is an open-source software for neonatal segmentation based on the algorithm proposed in Makropoulos et al. (2014). The algorithm performs atlas-based segmentation to divide the neonatal brain MRI into 87 regions. The different parts of the workflow are:

- Data pre-processing. The original MRI is brain-extracted to remove non-brain tissue and corrected for intensity inhomogeneity.
- Initial tissue segmentation. A spatio-temporal tissue atlas is registered to the brain MRI. The MRI is segmented into the different tissue types with an Expectation-Maximization scheme that combines an intensity model of the image with the tissue priors of the atlas.
- Structural atlas registration. Structural atlases (20 in total) are registered to the subject MRI with a multi-channel registration technique. The original intensity image and the GM probability map are used as different channels of the registration.
- Structure priors computation. The prior probability maps of the different structures are computed based on the local similarity of the transformed atlases with the input MRI.
- Label segmentation. The MRI is segmented into the different structures with a consequent Expectation-Maximization scheme.
- Post-processing. The segmented labels are merged in different granularities to further produce the final tissue segmentations and different hemispheres of the brain. Temporary files used for the computations are removed.

The software is used in collaboration between two teams, and potentially more when data from the developing HCP get publicly released. This workflow is a good example of common use cases evoked in introduction to this work. Here we are faced with two problems when we want to share the pipeline with collaborators: making the description portable from one system to another, and ensuring that the applications that form each stage can be re-executed on another environment.

A first excerpt from this workflow in Listing 5 shows how OpenMOLE interacts with CSV files to explore a fixed parameter space. The notion of samplings in OpenMOLE is flexible enough to traverse a parameter space described in a CSV file or using the more complex methods listed in Section 2.3.

**Listing 5:**
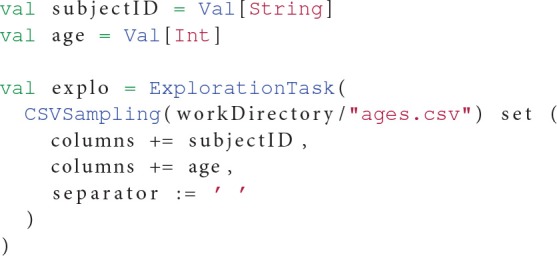
**CSV file exploration using samplings**.

A single CARE archive was prepared containing the necessary material for all the tasks of the original pipeline (available from Draw-EM's repository^20^). We have noticed that generating one archive per task generally leads to a large amount of duplicated binaries and shared libraries from one archive to another. When the different tasks of a pipeline share the same dependencies, it is thus more efficient to gather all of them in a unique archive. This strategy leverages OpenMOLE's file replication mechanisms better and reduces the amount of data transferred to remote environments.

The generated CARE archive is then integrated using CARETasks in the OpenMOLE workflow, and fed with input data files stored on the host machine. The command used in the original pipeline is reused as is to build the CARETask and accepts the parameters explored by the sampling in Listing 5. The resulting CARETask is presented in Listing 6.

**Listing 6:**
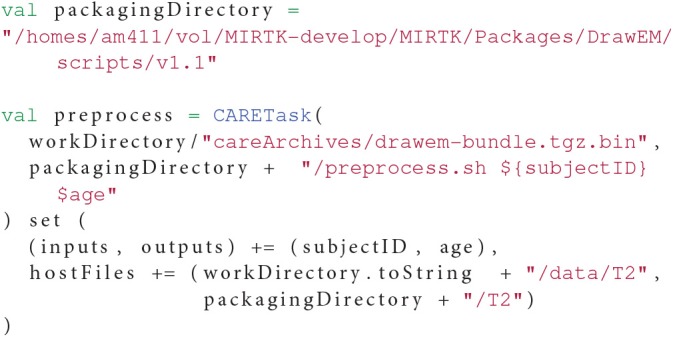
**The preprocessing CARETask extracted from the Draw-EM pipeline. Input data files are injected from the host system and parameters *subjectID* and *age* taken from the CSV sampling in Listing 5**.

As this pipeline is meant to be shared and labeled with a specific version, the fact that CARE archives are not as flexible as Docker turns from a drawback to an advantage as it makes it simpler to ship to the end-user. All the parameterizable parts of the pipeline are handled by the OpenMOLE script, and the pipeline can still be customized by inserting new tasks. Still, any user downloading the OpenMOLE workflow along with the associated CARE archives will be able to reproduce the same experiments that have been performed by the packager, or to reuse the same pipeline for further experiments and comparisons. It is important to note that the data necessary to run the pipeline are not included in the shipped CARE archives.

### 5.3. Advanced parameter tuning methods

This third workflow performs parameter optimization for cortical surface registration. In this example, cortical surface alignment is performed using the Multimodal Surface Matching tool (MSM) (Robinson et al., 2013); developed as part of the HCP to enable between subject alignment of multiple different types of cortical surface features (for example functional activations and cortical folding). Registration is optimized to maximize the ratio of feature similarity relative to surface warp distortions.

Here, we study a simplified version of the parameter optimization. The workflow consists in optimizing the value of nine parameters of the MSM tool for a fixed pair of subjects. The parameters explored can be found in Table 5.

**Table 5:** **Description of the parameters optimized for the MSM tool**.

**Parameter**	**Dimensionality**	**Range**	**Description**
Lambda	3	[0.00001, 100.0]	Weights the contribution of the regularizer relative to the similarity force.
sigma_in	3	[2; 10]	Sets the input smoothing: this changes the smoothing kernel's standard deviation
Iterations	3	[3; 5]	Controls the number of iterations at each resolution.

In order to find the optimal values for these parameters, we need to compute a fitness function that we will try to minimize using our methods. The fitness function estimates a distortion metric and is computed within its own OpenMOLE task as in Listing 7.

**Listing 7:**
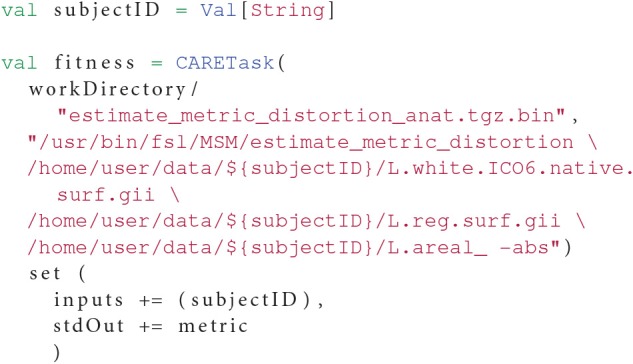
**The result metric is retrieved from the standard output *(the command lines have been simplified for the sake of readability)***.

Now, Listing 8 shows how the NSGA-II (Deb et al., 2002) could be initialized to optimize this problem in OpenMOLE.

**Listing 8:**
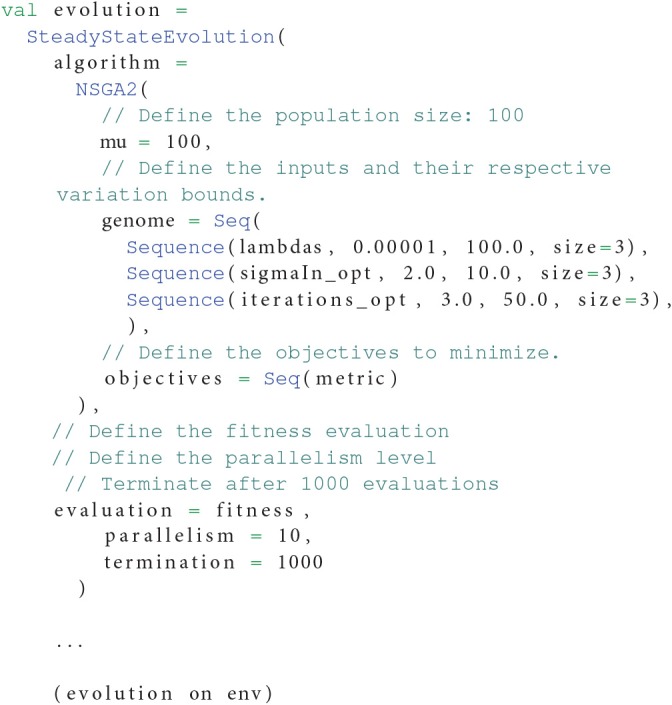
**Initialization of the NSGA-II algorithm with the parameters to optimize according to the fitness function from Listing 7. Multi-dimensional parameters are seamlessly handled by the algorithm**.

Advanced exploration methods are computationally greedy, but are well suited for parallelization on distributed computing environments. This exploration can also benefit from OpenMOLE's workload delegation by using the on keyword seen in Listing 4. This shows that exploration methods fit well in the OpenMOLE ecosystem and can benefit from the other components of the platform, such as the computing environments.

## 6. Conclusion

In this paper, we have shown the ability of the OpenMOLE scientific workflow engine to provide reproducible pipelines that can be shared and distributed on any Linux based environment.

We have seen that the OpenMOLE DSL provided a high-level description of experiments that can be shared and reused by scientists on any platform with a JVM. The newly added CARETask offers a solution to ensure Linux-based application can be packaged and re-executed seamlessly on another Linux host without the need to obtain administrator privileges. This criterion was necessary to target HPC environments, a de-facto choice to distribute experiments in the scientific world.

Extensions to the OpenMOLE DSL led to a fine integration of CARE in the framework. Archives only contain binaries and their dependencies, leaving the data to process to be injected in the archive's pseudo-filesystem at runtime from the dataflow. This results in a solution that can be shared from one machine to another, from the description of the pipeline to the applications composing its steps, with the single assumption that it will be re-executed on a Linux host.

Our experiments have reported successful re-executions with the distributed computing environments supported by OpenMOLE. In particular, Section 4 has shown that results obtained from a pipeline with complex software dependencies could be identically reproduced on an heterogeneous set of Linux computing environments.

Medical imaging pipelines were a perfect testbed for our solution, as they are composed of very diverse software tools. A description of case studies inspired from real-world medical imaging solutions has illustrated the suitability of the solution to handle reproducible medical imaging experiments at large scale. Problems such as enabling finer grain parallelism in pipelines, enhancing pipeline sharing with the community, and automatic parameter tuning are three of the concerns that can be encountered by researchers tackling large-scale medical imaging studies. We have addressed these topics through OpenMOLE implementations of three inhouse neuroimaging pipelines. They have showcased various features of the OpenMOLE platform that can help sharing and reproducing pipelines.

OpenMOLE, as well as all the tools forming its ecosystem, are free and open source software distributed under the Affero General Public License version 3 (AGPLv3). This allows anyone to contribute to the main project, or build extensions on top of it.

Future releases of the OpenMOLE platform will strengthen the support of cloud computing environments, with a particular attention given to Amazon EC2. As major datasets become publicly available in the Amazon cloud, moving neuroimaging studies to the cloud is necessary to explore whole datasets. Reproducible OpenMOLE workflows are a valuable addition to the set of tools available to the community in order to set up ambitious experiments.

## Author contributions

JP has led this work, drafted the initial version of the manuscript, and is an active contributor to the OpenMOLE project. RR is the leader of the OpenMOLE project and a main developer. ML is a main developer of the OpenMOLE project and has created the graphical user interface. ER, AM, and SP are the original authors of the pipelines presented as case studies. DR has taken part in the inception and conception phases of this work. All authors have revised and agreed on the content of the manuscript.

## Funding

The research leading to these results has received funding from the European Research Council under the European Union's Seventh Framework Programme (FP/2007-2013)/ERC Grant Agreement no. 319456.

### Conflict of interest statement

The authors declare that the research was conducted in the absence of any commercial or financial relationships that could be construed as a potential conflict of interest.

## Footnotes

^1^http://humanconnectome.org/.

^2^http://fsl.fmrib.ox.ac.uk.

^3^http://www.fastr.eu/.

^4^https://wiki.galaxyproject.org/.

^5^https://taverna.incubator.apache.org/introduction/taverna-features.

^6^http://pipeline.loni.usc.edu/explore/features/.

^7^https://code.kepler-project.org/code/kepler-docs/trunk/outreach/documentation/shipping/2.5/UserManual.pdf.

^8^https://pegasus.isi.edu/documentation/execution_environments.php.

^9^https://github.com/adraghici/openmole/tree/aws-env.

^10^https://github.com/openmole/gridscale.

^11^http://www.openmole.org/current/Documentation_Language_Samplings.html.

^12^https://en.wikipedia.org/wiki/Sobol_sequence.

^13^http://en.wikipedia.org/wiki/Latin_hypercube_sampling.

^14^https://github.com/pgbovine/CDE/commit/219c41590533846de12d7c5cca3f34aac471aae7., *last accessed 12-nov-16*.

^15^https://nilearn.github.io/auto_examples/02_decoding/plot_haxby_different_estimators.html, *last accessed on 12-nov-16*.

^16^https://github.com/openmole/openmole-market.

^17^https://github.com/openmole/frontiers2016.

^18^https://db.humanconnectome.org.

^19^https://github.com/MIRTK/DrawEM.

^20^https://github.com/MIRTK/DrawEM/blob/c98022a5b78ee99bef5d329fc23f57f9c15b1a5f/pipelines/neonatal-pipeline-v1.1.sh.
